# Diagnostic Usefulness of Cell Block Samples in Fluid Overload‐Associated Large B‐Cell Lymphoma

**DOI:** 10.1002/dc.70198

**Published:** 2026-08-11

**Authors:** Hiroyuki Okanishi, Hiroko Kuwabara, Hiroko Nakayama, Mayo Odaka, Kozue Ariga, Isako Kataoka, Mari Tomiuka, Mayumi Uragami, Chihiro Deguchi, Shizuka Ono, Reika Takeda, Yoshitaka Kurisu, Yoshinobu Hirose, Yuuki Kinoshita, Taiji Yokote

**Affiliations:** ^1^ Division of Diagnostic Pathology Osaka Medical and Pharmaceutical University Hospital Osaka Japan; ^2^ Division of Hematology Osaka Medical and Pharmaceutical University Hospital Osaka Japan

**Keywords:** cell block, conventional smear, fluid cytology, FO‐LBCL, lymphoglandular body

## Abstract

Fluid overload‐associated B‐cell lymphoma (FO‐LBCL) is a B‐cell neoplasm that presents as serous effusions without detectable tumor masses and Kaposi sarcoma‐associated herpesvirus (KSHV)/human herpesvirus 8 (HHV8) infection. Here, we report a case of an 81‐year‐old female patient with FO‐LBCL (Stage IVA) who presented with pericardial and pleural effusions. The patient had no underlying medical condition that could lead to fluid overload. Cytological examination of both effusions revealed medium‐ to large‐sized lymphoid cells with high nucleocytoplasmic ratios, irregular nuclei, dense chromatin, prominent nucleoli, mitotic figures, basophilic cytoplasm, and lymphoglandular bodies. A cell block of the pleural fluid effusion showed that atypical lymphoid cells were positive for CD20 and CD79a, and negative for CD3, KSHV/HHV‐8, and Epstein–Barr virus. The Ki‐67 labeling index was 95%. The patient underwent systemic chemotherapy and achieved complete remission without pleural or pericardial recurrence during the 3‐year follow‐up period. As FO‐LBCL is diagnosed based on cytological material, together with clinical and imaging findings, immunohistochemical analysis of the cell block is useful for confirming the diagnosis and selecting tailored therapies.

## 1 Introduction

Fluid overload‐associated B‐cell lymphoma (FO‐LBCL) is a very rare B‐cell neoplasm, with < 100 reported cases, presenting as serous effusions without detectable tumor masses and Kaposi sarcoma‐associated herpesvirus (KSHV)/human herpesvirus 8 (HHV8) infection [1]. Lymphomas mainly involving the body cavity include primary effusion lymphoma (PEL) [2] and diffuse large B‐cell lymphoma associated with chronic inflammation (CI‐DLBCL) [3]. Although these lymphomas are diagnosed using cytological specimens, immunophenotyping analyses and viral infections are essential for their differentiation. PEL lacks B‐cell markers such as CD20 and CD79a, is consistently associated with KSHV/HHV8, and is usually coinfected with the Epstein–Barr virus (EBV) [2]. CI‐DLBCL expresses B‐cell markers and is associated with EBV [3]. FO‐LBCL expresses B‐cell markers and is not associated with KSHV/HHV8 [1]. Therefore, evidence of B‐cell markers and viral infection in tumor cells is important to differentiate between them. Here, we describe the case of an 81‐year‐old woman diagnosed with FO‐LBCL, which was confirmed by cell block analysis. Moreover, because there are few reports describing the cytology of FO‐LBCL, cytological characteristics were retrospectively reviewed.

## 2 Case Report

An 81‐year‐old Japanese woman presented to a neighboring hospital with general fatigue. The patient had paroxysmal atrial fibrillation and underwent abdominal aortic aneurysm repair using an artificial graft at the age of 75. Tests for human immunodeficiency virus (HIV), hepatitis B virus, and hepatitis C virus antibodies were negative. Computed tomography revealed pericardial and pleural effusions (Figure 1), and the patient was admitted to Osaka Medical and Pharmaceutical University Hospital. Hematological evaluation revealed that lactate dehydrogenase and soluble interleukin‐2 receptor levels were 557 U/L (normal range: 124‐222) and 622 U/mL (normal range: 121‐613), respectively. Pericardiocentesis and thoracocentesis were performed, samples were centrifuged, and wet and dry smears were prepared for Papanicolaou (wet smear) and May–Grunwald Giemsa (MGG) (dry smear) staining. Cytologically, smears of both effusions showed abundant cellularity, consisting of medium‐ to large‐sized lymphoid cells with high nucleocytoplasmic (N/C) ratios, irregular nuclei, dense chromatin, and prominent peripheral nucleoli (Figure 2A,C). Lymphoglandular bodies (LGBs) in the background and mitotic figures were observed (Figure 2A,B). MGG‐stained smears of the right‐sided pleural fluid showed tumor cells with the basophilic cytoplasm (Figure 2D). Cell block sections of the right pleural fluid revealed that atypical lymphoid cells were positive for CD20 and CD79a (Figures 3B,C), and negative for CD3 and KSHV/HHV8. In situ hybridization for EBV‐encoded small RNA yielded negative results, with a Ki‐67 labeling index of 95% (Figure 3D). Both effusion samples revealed heavy chain immunoglobulin rearrangements. Examination of the bone marrow revealed no lymphoma cells, and no solid mass was identified. Based on these findings, the diagnosis of FO‐LBCL was made. The patient was treated with polatuzumab Vedotin (Pola) plus rituximab, cyclophosphamide, doxorubicin, and prednisone (Pola‐R‐CHP) chemotherapy, because vincristine could not be administered due to a history of adhesive ileus. The patient has been lymphoma‐free for 3 years.

**FIGURE 1:**
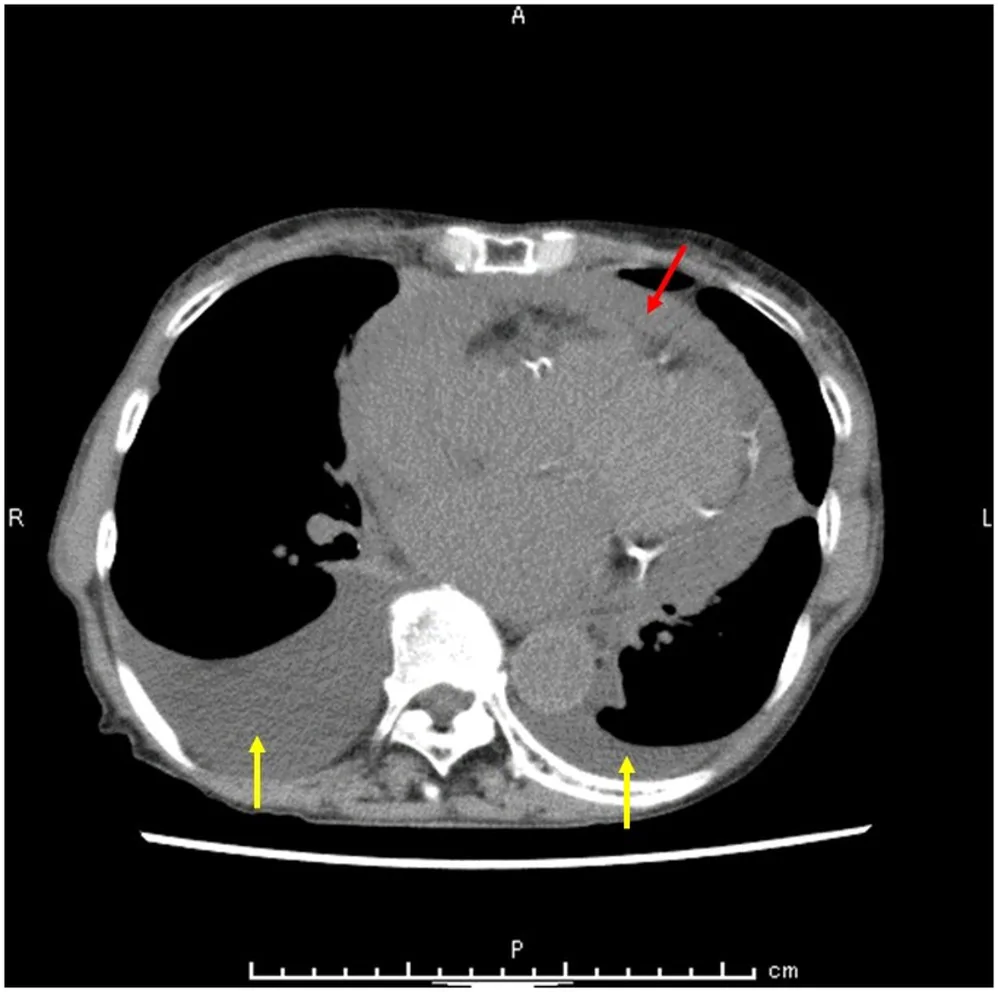
Chest computed tomography showing pericardial (see red arrow) and pleural effusions (see yellow arrows).

**FIGURE 2:**
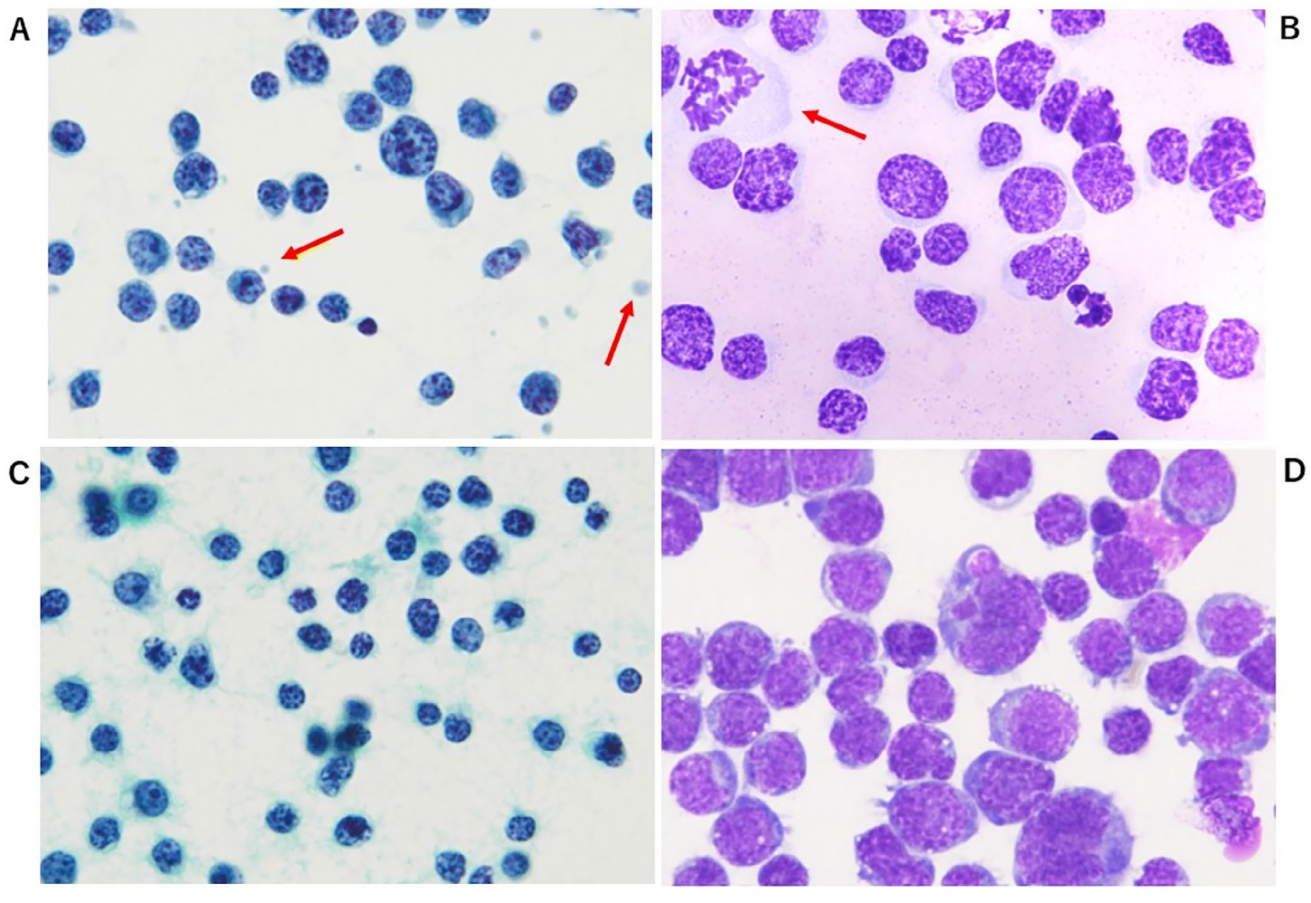
Smears of the pericardial (A, B) and right pleural effusions (C, D). (A) Medium‐to‐large lymphoid cells with a high N/C ratio and prominent nucleoli. Lymphoglandular bodies (A, see arrows) (Papanicolaou stain ×1000). (B) Lymphoid cells with irregular nuclei and mitotic figures (see arrow) (May–Grunwald Giemsa stain ×1000). (C) Medium‐to‐large lymphoid cells with a high N/C ratio and prominent nucleoli (Papanicolaou stain ×1000). (D) Lymphoid cells with basophilic cytoplasm (May–Grunwald Giemsa stain ×1000).

**FIGURE 3:**
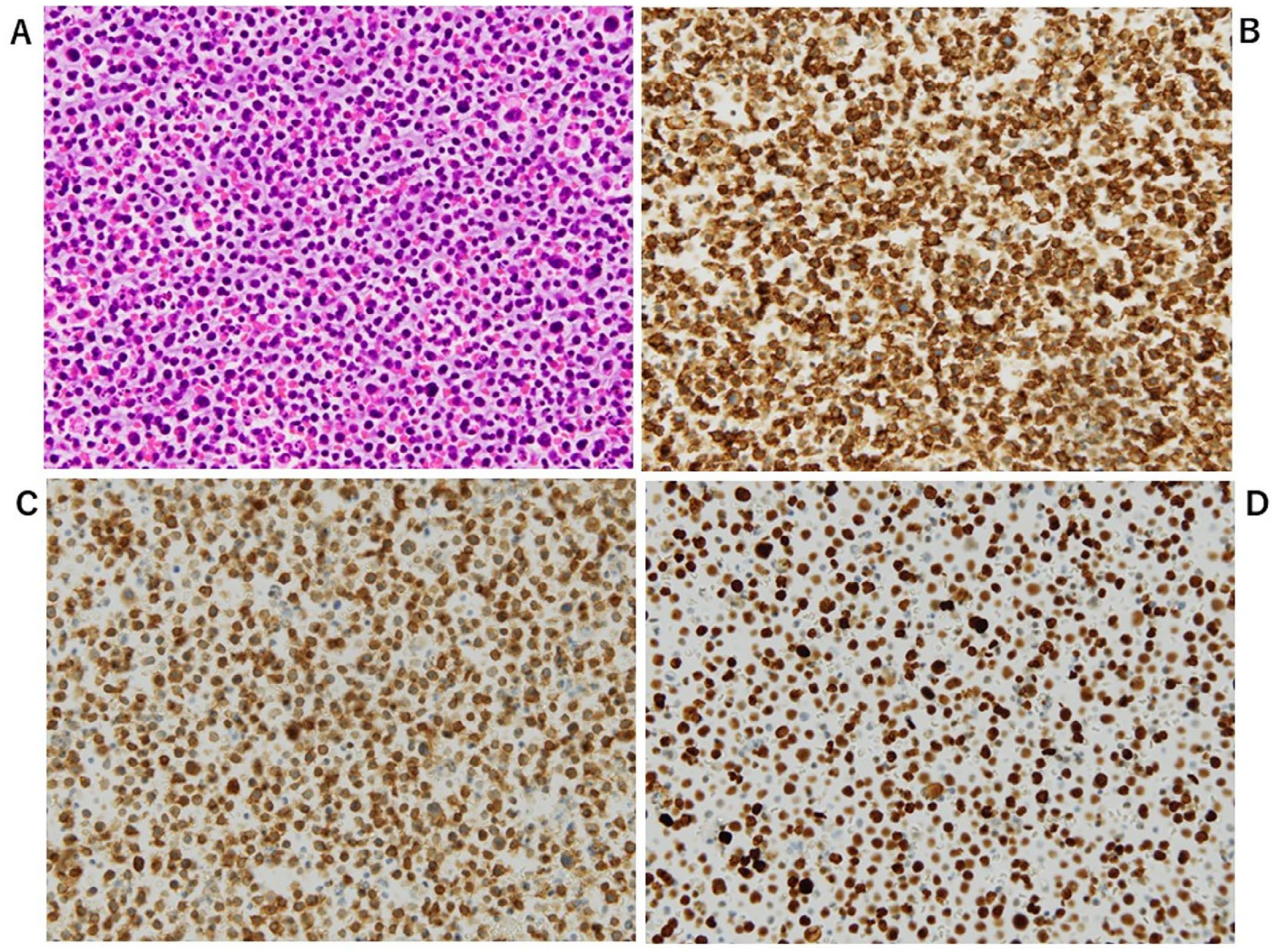
Cell block section of the right‐sided pleural effusion. Atypical lymphocytes were positive for CD20 (B) (×400) and CD79a (C) (×400). Ki‐67 labeling index was 95% (D) (×400). (A) Hematoxylin and eosin staining (×400).

## 3 Review of Clinicocytological Features of FO‐LBCL

We reviewed the cytological characteristics of 10 patients with FO‐LBCL, including the present case, which revealed a detailed cytological description, as well as immunohistochemical and viral findings (Table 1) [4, 5, 6, 7, 8, 9, 10]. The patients were elderly (59–82 years, median age: 73.8 years), and included six males and four females. The sites of involvement were the pleura (seven cases), pericardium (three cases), and abdominal cavity (two cases). All patients exhibited high N/C ratios, irregular nuclei, prominent nucleoli, mitotic figures, and chromatin abnormalities. The Ki‐67 labeling index was high (60%–95%), and mitotic figures were frequent. LGBs were not described in previous nine cases.

**TABLE 1:** Clinicocytological features of FO‐LBCL.

Patient	Age (years)	Sex	Location	High N/C ratio	Irregular nuclei	Chromatin	Prominent nucleoli	Mitosis	Ki‐67 index	References
1	69	M	Pleural effusion	NA	+	NA	NA	+	> 80%	[4]
2	70	M	Ascites	NA	NA	NA	NA	NA	60%	[5]
3	80	M	Pleural effusion	+	+	NA	NA	NA	NA	[6]
4	73	F	Pericardial effusion	+	NA	Vacuolated	NA	+	70%	[7]
5	75	M	Pleural effusion	NA	NA	Coarse, granular	+	+	80%	[7]
6	72	M	Ascites, pleural effusion	NA	NA	Fine	NA	NA	> 90%	[8]
7	82	F	Pleural effusion	NA	+	Hyperchromatic	+	+(atypical)	80%	[9]
8	59	F	Pericardial effusion	NA	+	Hyperchromatic	NA	NA	NA	[9]
9	77	M	Pleural effusion	NA	+	open	+	NA	NA	[10]
Present case	81	F	Pericardial effusion, pleural effusion	+	+	Hyperchromatic	+	+	95%	

Abbreviation: NA: not available.

## 4 Discussion

FO‐LBCL is a B‐cell neoplasm that presents as a serous effusion without a solid mass, and its diagnosis is commonly based on cytological materials, as well as clinical and imaging findings. Half of patients with FO‐LBCL have underlying medical conditions leading to fluid overload [1], and the present patient had no such conditions. The tumor cells of the present patient exhibited high N/C ratios, irregular nuclei, chromatin abnormalities, prominent nucleoli, and mitotic figures. The cytoplasm was basophilic, and LGBs were observed in the background. These findings were consistent with those of large B‐cell lymphoma [11], and the other nine FO‐LBCL cases showed almost the same findings [4, 5, 6, 7, 8, 9, 10]. LGBs are stripped cytoplasmic fragments in the background of smears that can be observed in lymphoma but not in carcinoma [12]. In particular, these bodies are observed in Burkitt lymphoma and high‐grade B‐cell lymphoma with MYC and BCL2 rearrangements (double‐hit lymphoma), which have high proliferation indices [13, 14]. The present case and reviewed FO‐LBCL cases showed a high Ki‐67 labeling index. The reviewed 9 cases revealed no LGBs, but Case 3 showed necrotic substances, suggesting LGBs [6]. LBGs may be an adjunct indicator for the diagnosis of FO‐LBCL, however, further case studies are required.

Body cavity‐based lymphomas include PEL and CI‐DLBCL. Immunophenotypic and viral infection analyses are essential for differentiating tumors from FO‐LBCL. PEL is the proliferation of mature B cells that show heavy chain immunoglobulin rearrangements and lack pan‐B‐cell markers (CD20 and CD79a) [1]. It is consistently associated with KSHV/HHV8 infection and is usually coinfected with EBV and HIV. In the present case, the tumor cells were positive for CD20 and CD79a and negative for KSHV/HHV8, consistent with FO‐LBCL. Pan‐B‐cell markers and KSHV/HHV8 infection are key factors in differentiating FO‐LBCL from PEL. KSHV/HHV8 drives B‐cell lymphoid proliferation and lymphoma, including PEL, in immunodeficiency patients [15]. PEL has a relatively poor prognosis compared to FO‐LBCL [2], and differentiation between these diseases is important. Another differential diagnosis is CI‐DLBCL, which is characterized by CD20 positivity and EBV infection. In this case, lymphoma cells were negative for EBV. For these immunohistochemical studies, cell block analysis of fluid samples is useful. Peritoneal washing cytology has become widely performed for staging of gynecological malignancies, and cell block immunohistochemistry is very effective compared to conventional cytology and liquid‐based cytology [16]. A cell block of pleural effusion was used to diagnose FO‐LBCL [4].

While immunophenotypic analysis is necessary for treatment. Systemic chemotherapy, such as rituximab, cyclophosphamide, doxorubicin, vincristine and prednisone (R‐CHOP), is the standard treatment for B‐cell lymphomas, including FO‐LBCL. Rituximab is a monoclonal antibody that targets CD20‐positive B‐cells, and confirmation of CD20 expression in lymphoma cells is necessary for treatment. Furthermore, Pola‐R‐CHP therapy, which was administered in the present case, is more effective than conventional R‐CHOP [17]. Pola is an antibody‐drug conjugate directed against CD79b, and CD79a and CD79b form a heterodimeric signaling component of the B‐cell receptor. Therefore, immunohistochemical studies on both CD20 and CD79a may be required.

## 5 Conclusion

FO‐LBCL is characterized by the primary presentation of serous cavity effusion in the absence of solid masses. Since the diagnosis of FO‐LBCL is based on cytological material and clinical and imaging findings, immunohistochemical analysis of a cell block sample is useful for confirming the diagnosis and selecting tailored therapies.

## Funding

The authors have nothing to report.

## Disclosure

The authors have nothing to report.

## Conflicts of Interest

The authors declare no conflicts of interest.

## Data Availability

The data that support the findings of this study are available on request from the corresponding author. The data are not publicly available due to privacy or ethical restrictions.
